# The Effects of Gold Kiwifruit Intake Timing with or without Pericarp on Postprandial Blood Glucose Level

**DOI:** 10.3390/nu13062103

**Published:** 2021-06-19

**Authors:** Yutaka Inoue, Yukari Kitani, Satoshi Osakabe, Yukitoshi Yamamoto, Isamu Murata, Ikuo Kanamoto

**Affiliations:** 1 Laboratory of Drug Safety Management, Faculty of Pharmacy and Pharmaceutical Sciences, Josai University, 1-1 Keyakidai, Sakado 3500295, Saitama, Japan; yy15092@josai.ac.jp (Y.K.); yy15067@josai.ac.jp (S.O.); yy15282@josai.ac.jp (Y.Y.); ismurata@josai.ac.jp (I.M.); i.kanamoto@gmail.com (I.K.); 2 Laboratory of Nutri Pharmacotherapeutics Management, Faculty of Pharmacy and Pharmaceutical Sciences, Josai University, 1-1 Keyakidai, Sakado 3500295, Saitama, Japan

**Keywords:** gold kiwifruit, postprandial blood glucose, intake timing, pericarp

## Abstract

The purpose of this study was to examine how gold kiwifruit pericarp (pericarp is defined as the skin of the fruit) consumption and the timing thereof affect the postprandial blood glucose profile. The study was conducted on twelve healthy volunteers (six men and six women). According to our results, the simultaneous intake of gold kiwifruit with bread and the prior intake of gold kiwifruit evidently suppressed the postprandial blood glucose elevation compared with exclusive bread intake. There was no significant difference in postprandial blood glucose changes between the ingestion of gold kiwifruit pericarp and pulp and that of gold kiwifruit pulp only. The highest postprandial blood glucose elevation was suppressed by 27.6% and the area under the blood glucose elevation curve by 29.3%, even with the exclusive ingestion of gold kiwifruit pulp. We predicted that the ingestion of both the pericarp and pulp of gold kiwifruit would reduce the postprandial blood glucose elevation to a greater extent than that of gold kiwifruit pulp only; however, there was no significant difference between the two. These results indicate that gold kiwifruit consumption significantly suppresses the postprandial blood glucose elevation regardless of pericarp presence or absence and the timing of ingestion.

## 1. Introduction

According to the 2016 National Health and Nutrition Survey, the total number of people with diabetes and those in the preparatory group for diabetes is reported to be approximately 20 million [1]. Diabetes causes various complications and increases the risk of developing cerebrovascular and heart diseases, which are the leading causes of death in Japan [2]. It has been suggested that a rapid rise in postprandial blood glucose levels promotes the progression of various lifestyle-related diseases; therefore, the consumption of foods that moderates the postprandial blood glucose elevation is recommended. Diabetes etiology is strongly associated with lifestyle and dietary changes [3], and there is emerging interest in carbohydrate restriction to control the dietary blood glucose elevation [4,5] and promotion of low glycemic index (GI) foods [6].

Kiwifruit, scientifically known as *Actinidia deliciosa*, is cultivated in temperate to subtropical regions and is a fruit that can be obtained at low cost throughout the year. It is of high value because it contains abundant nutrients, such as dietary fiber, vitamin C, and potassium. In addition, it can be eaten with ease when cut in half, is considered a “busy morning” snack, and is a nutritionally suitable fruit for people without sufficient nutrient intake such as an increased breakfast skipping rate, excessive dieting, and excessive dependence on processed foods and specific foods. The gold kiwifruit is more nutritious than the green variety, considering that it contains approximately twice as much vitamin C (161 mg/100 g) [7]. A study by Mishra et al. [8] suggested that when 1/3 of the carbohydrate content of breakfast cereal is replaced with kiwifruit and consumed at the same time daily, the postprandial blood glucose elevation is suppressed as compared to that resulting from breakfast cereal alone. Kiwifruit has a brown pericarp and is covered with hairy fibers. The Japanese population generally consumes peeled kiwifruit, whereas New Zealanders habitually consume it with its pericarp. Since the pericarp of kiwifruit contains more polyphenols than the flesh [9], it is possible that consuming the flesh and pericarp together may have a positive effect on the postprandial blood glucose profile. In other words, a confirmation of the impact of kiwifruit pericarp on the suppression of the blood glucose elevation in the postprandial blood glucose profile may contribute to the management of lifestyle-related diseases, as a recommended dietary and nutritional guidance component.

It is used to predict postprandial blood glucose responsiveness and promote the development and provision of foods and meals with a moderate increase in blood glucose. That is, an in vitro evaluation method (glucose release rate (GR)) that imitates the physical and biochemical digestive processes of humans has recently been developed [10]. The application of the GI measurement of foods consumed by human subjects is prone to large measurement errors, such as individual differences, and incurs research time and high costs. Furthermore, the activity restrictions, large intake of glucose solution, and blood sampling associated with the GI measurement may cause stress in subjects. We reported that drinking vegetable juice before or with a carbohydrate-based meal attenuated the elevation of postprandial blood glucose levels [11]. Moreover, we found that drinking approximately 200 mL of vegetable juice 30 min before eating was the most effective way to suppress the elevation of postprandial blood glucose levels. Hence, blood glucose levels were also measured in two different groups of golden kiwifruit (one that simultaneously ingested bread and gold kiwifruit and another where kiwifruit was ingested 30 min before bread).

Therefore, an attempt to predict the postprandial blood glucose elevation by incorporating the GR method into the evaluation as an alternative to the GI method is of paramount importance. Therefore, we focused on the gold kiwifruit and examined blood glucose, GR, and satiety in order to establish the differences in postprandial blood glucose profiles after kiwifruit pericarp ingestion, with and without pulp, between a group that simultaneously ingested bread and gold kiwifruit and another that ingested gold kiwifruit 30 min prior to bread consumption.

## 2. Materials and Methods

### 2.1. Subjects

Blood glucose measurements and satiety tests were performed on 15 healthy adults (6 men and 9 women). In this study, the exclusion criteria were as follows: (1) women undergoing menstruation, (2) food allergies to the bread and kiwifruit used in this study, (3) drug treatment, (4) alcohol hypersensitivity, (5) no experience with consuming kiwifruit, and (6) abnormal liver or renal function. The results of 12 subjects (6 men and 6 women) were eventually used for data analysis in this study because data outside the mean ± 2 standard deviation (SD) range of the area under the blood glucose elevation curve were rejected, of which 3 people had data outside this range. This study was approved by the Josai University Ethics Review Board for Medical and Health Research Involving Human Subjects (approval number: 2018-11A). It was conducted in accordance with the principles of the Declaration of Helsinki. The purpose, content, and safety of the study were fully explained to all subjects, and the study was conducted with the consent of the subjects. The baseline characteristics of the 12 subjects are presented in Table 1. The baselines (height and weight) of the subjects were measured before they participated in the study (Table S1). HbA1c is measured in the fasting state.

### 2.2. Test Meals and Reagents

The ripe gold kiwifruit was provided by Zespri International Japan Co., Ltd. “Honjikomi bread” was purchased from Fujipan Co., Ltd., and invertase from Merck KgaA (Darmstadt, Germany). Pepsin, pancreatin, and other reagents were acquired from Fujifilm Wako Pure Chemical Industries, Ltd. Table 2 displays each nutritional component of the test meal.

### 2.3. Test Protocol

The subjects’ age, height, and weight were initially assessed. Study subjects refrained from strenuous exercise from the day before the test, and on the test day, activity was limited to quiet standing or sitting until the end of the test. In addition, subjects fasted from 21:00 h on the day before the test until the start of the test (water intake was permissible). Subjects were placed in the following six groups: (1) treatment group B: bread (B, 109.1 g) intake, (2) BPK treatment group: simultaneous consumption of B (79.9 g) and gold kiwifruit pulp (PK, 100 g), (3) BWK treatment group: simultaneous consumption of B (79.9 g) and gold kiwi pericarp/pulp (WK, 92.4 g), (4) B30B treatment group: consumption of B (79.9 g) 30 min after ingesting B (29.2 g), (5) PK30B treatment group: consumption of B (79.9 g) 30 min after ingesting PK (100 g), and (6) WK30B treatment group: consumption of B (79.9 g) 30 min after ingesting WK (92.4 g). The interval between each test was approximately 3–7 days. The total amount of available carbohydrates was set to 50 g in all treatment groups, and water intake during the test period was limited to 200 mL (water or plain hot water). All participants completed the following treatments in a randomized order crossover method. On the day of the test, blood glucose measurements were performed on the B, BPK, and bled-BKW groups before ingestion (0 min) as well as 15, 30, 45, 60, 90, 120, and 180 min after test-meal intake. Blood sampling in the B30B, PK30B, and WK30B treatment groups was performed before intake (−30 min); 15 min after intake of B, PK, and WK (−15 min); before intake of B (0 min); and 15, 30, and 45 min after intake. Blood glucose levels were measured at 60, 90, 120, and 180 min. In addition, the degree of satiety was evaluated concurrently with the blood glucose measurement.

### 2.4. Blood Sampling and Blood Glucose Measurement

Blood was collected by fingerprick blood samples which were taken using a puncturing device (Medisafe Fine Touch, Terumo Corporation, Tokyo, Japan). Blood glucose was measured using a chip (Glutest Neo sensor, Sanwa Kagaku Kenkyusho Co., Ltd., Nagoya, Aichi, Japan) and a self-test glucose measuring device (Glutest Neo Alpha, Sanwa Kagaku Kenkyusho Co., Ltd., Nagoya, Aichi, Japan). Two fingerprick blood samples were taken at each time point and the average reported. If the difference fell outside 10 mg/dL between the two blood glucose values obtained, we remeasured the blood glucose levels until a third measure was taken within 10 mg/dL.

### 2.5. Satiety Score

The satiety level of kiwifruit intake was measured at the same time as the blood sampling, and the subject was asked to rate the level of satiety using a numerical rating scale with 11 levels ranging from 0 (very hungry) to 10 (no more hunger).

### 2.6. Measurement of Glucose Releasing Rate (GR)

To evaluate the digestibility of carbohydrates in vitro, a GR measurement was performed with (1) B, (2) PK, (3) WK, (4) BPK, and (5) BWK samples. The bread and gold kiwifruit in each group were cut into small pieces, and water was added and ground with a meat grinder (BOSCH, MUZ4FW3). The composition of each sample was as follows: B: bread (109.1 g) and 110 g of water; PK: gold kiwifruit pulp (100 g); WK: gold kiwifruit pericarp/pulp (100 g); BPK: bread (79.9 g) and PK (100 g); and BWK: bread (79.9 g) and WK (92.4 g). After grinding with a meat grinder, 10 g of each sample and 60 g of water were placed in a storage bottle; 5 mL of pepsin-hydrochloric acid solution were subsequently added, and the mixture stirred. Thereafter, the sample solution was allowed to stand in a thermostat chamber (TAITEC, BR-43FL) at 37 °C for 30 min. Subsequently, bread containing 10 mL of 0.39 M tri-Na phosphate solution, 5 mL of 2.0 M sodium chloride solution, 5 mL of 20% sorbic acid K solution, and Invertin MERCK (Merck KgaA Darmstadt Germany) was obtained. Five milliliters of a solution of Cleatin (FujiFilm Wako) (300 U/mL) was added, and the mixture was shaken in a constant temperature bath at 37 °C for 20 min (shaking width, 22 mm, and shaking number, 160 rpm). After 20 min, 3 mL of the dispersion were collected and filtered through a 5 µm membrane filter (Tomsic, TITAN3-30 NYLON, Tokyo, Japan), and 100 µL of the filtrate were used as the test solution for 20 min. The test solution was stored frozen at −80 °C for 20 min. After collecting 3 mL, the storage bottle was returned to a constant temperature bath and shaken at 37 °C for 16 h. After 16 h, a sample was collected using the same method as that for the 20 min test solution, and this was used as the 16 h test solution. The 20 min and 16 h test solutions were placed in ice-cold water, and 1900 µL of 50 mM phosphate buffer and 500 µL of rat small-intestinal acetone powder (Sigma-Aldrich, St. Lowis, MO, USA) extraction solution were added. The solution was reacted at a storage temperature of 37 °C for 40 min. After allowing the sample solution to stand for 40 min, 2500 µL of 0.2% acarbose solution were added, and the mixture was stirred with a vortex mixer to stop the enzymatic reaction. For each glucose concentration measurement, the glucose CII-Test Wako (Fuji Film Wako Pure Chemical Industries, Ltd., Tokyo, Japan) was used for the 20 min and 16 h test solutions. The GR value was calculated using Equation (1):

(1) $$\mathrm{GR}=\frac{20-\min\;\mathrm{test}\;\mathrm{solution}\;\mathrm{glucose}\;\mathrm{concentration}\;\left(µg/\mathrm{mL}\right)}{16-h\;\mathrm{test}\;\mathrm{solution}\;\mathrm{glucose}\;\mathrm{concentration}\;\left(µg/\mathrm{mL}\right)}\times 100$$

### 2.7. PH Measurement

The B, PK, WK, BPK, BWK, and gold kiwifruit were ground using a meat grinder (BOSCH, MUZ4FW3) and had their pH measured using HORIBA LAQUA twin-pH-22B.

### 2.8. Data Analysis

The value obtained by subtracting the blood glucose level (BG) immediately before breakfast intake from that over time was defined as ΔBG. The baseline corresponds to the fasting blood glucose level. The maximum ΔBG after ingestion of the test meal was defined as ΔBGmax, and the time to reach ΔBGmax was defined as Tmax. The incremental area under the curve (iAUC) of blood glucose was calculated using the trapezoidal rule with ΔBG.

### 2.9. Statistical Analysis

Data are expressed as mean ± standard error of the mean (SEM). Groups were compared using one-way analysis of variance (Statcel4), GR measurements using Tukey’s test, and blood glucose levels using Dunnett’s test for multiple comparisons. *: *p* < 0.05 and **: *p* < 0.01 were considered statistically significant.

## 3. Results

### 3.1. Blood Glucose Level

**Figure 1:**
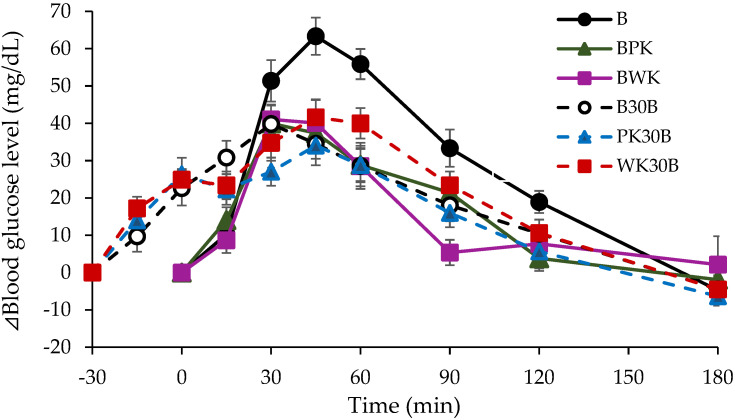
Mean difference between groups in ΔBlood Glucose. Data are shown as mean ± SEM (*n* = 12). No significant difference: Each treatment group. *p* < 0.05 vs. Bread, 30–180 min (Dunnett’s test).

The blood glucose profile, ΔBGmax, Tmax, and iAUC results for each test group are shown in Figure 1 and Table 3. The rate of attenuation in ΔBGmax and iAUC in each group compared to that in group B is summarized in Table 4. The postprandial blood glucose elevation was significantly suppressed in the BPK, BWK (simultaneous intake of gold kiwifruit and bread), PK30B, and WK30B groups (intake of bread after ingestion of gold kiwifruit) as compared with that in group B (bread alone). The postprandial glucose response was attenuated in the BPK group by 27.6% in the BPK group and 25.1% in the BWK group compared with that in group B, showing significant suppression (*p* < 0.05). In the B30B, PK30B, and WK30B groups, ΔBGmax was significantly (*p* < 0.01) lower than that in group B. The iAUC was significantly lower (*p* < 0.05) in the BPK group than in group B and also showed significantly lower values (*p* < 0.01) in the BWK and PK30B groups. On comparing the BWK (simultaneous intake of bread and kiwifruit pericarp and pulp) and BPK (intake of only bread and gold kiwifruit pulp) groups, there was no significant difference in ΔBGmax, with or without pericarp intake.

### 3.2. Satiety Test

**Figure 2:**
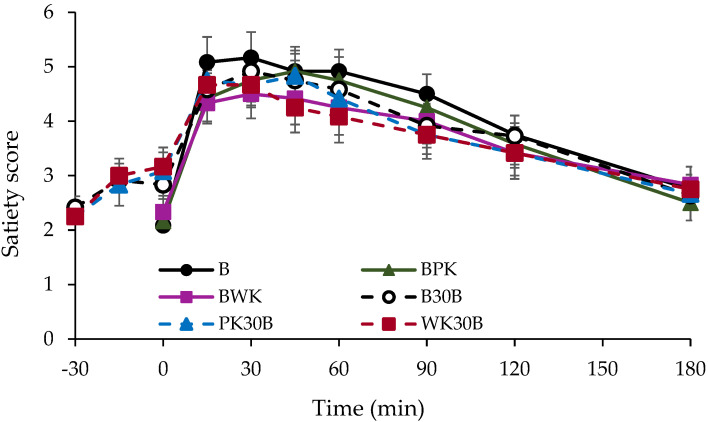
Changes in satiety score. Data are presented as mean ± SEM.

Figure 2 presents the satiety test results for each group. No significant difference in satiety was observed between the test groups 180 min after test-meal ingestion. Satiety did not attenuate in the BPK and BWK groups, in which the carbohydrate content of bread was replaced with gold kiwifruit. In addition, no significant difference in the feeling of fullness was observed between the different intake groups, that is, those with and those without pericarp intake.

### 3.3. Glucose Releasing Rate Test

GR test results are displayed in Table 5; the GR values were 77.5 and 83.8 in the BPK and BWK groups, respectively, with no significant difference compared to 78.3 in group B.

### 3.4. PH Measurement

The sample pH values were measured and found to be 3.3, 3.5, 3.6, and 3.8 for PK, WK, BPK, and BWK, respectively. The pH values of WK and BWK with pericarp were approximately 0.2 times higher than those of PK and BPK without pericarp.

## 4. Discussion

This study demonstrated that the simultaneous intake of gold kiwifruit with bread (BPK and BWK groups) and prior intake (PK30B and WK30B groups) both refer to the suppressed postprandial blood glucose elevation compared to the intake of bread alone (group B). In a study by Lubransky et al. [12], ΔBGmax and iAUC reportedly attenuated by 11% and 30%, respectively, when 40% of the total carbohydrate mass in 65 g of porridge was replaced with kiwifruit, which was ingested 30 min before porridge consumption. In our study, ΔBGmax and iAUC were reduced by 27.6% and 29.3%, respectively, in the BPK group, in which approximately 37% of the bread carbohydrate content was replaced with 100 g of PK and consumed simultaneously with bread, suggesting that the inclusion of gold kiwifruit pulp in a meal is sufficient to suppress the postprandial blood glucose elevation. The subdued rate of gastric emptying is considered one reason for the suppression of the postprandial blood glucose elevation by gold kiwifruit. Gold kiwifruit is rich in organic acids, such as citric acid, quinic acid, and malic acid [13], and dietary fiber, which slows down the rate of gastric emptying [14,15,16], thus suppressing the postprandial blood glucose elevation compared to bread alone (group B). A study by Mishra et al. reported suppressed postprandial blood glucose levels in the kiwifruit combination group, in which kiwifruit was combined with wheat cereal, compared to those in the group consuming wheat cereal alone [17]. A possible mechanism underlying the suppression of the postprandial blood glucose elevation after the simultaneous consumption of wheat cereal and kiwifruit might have been delayed gastric emptying [18] and ileal digestion through delayed pH adjustment in the stomach and duodenum affected by the organic acids in kiwifruit. The phenolic compounds in kiwifruit potentially inhibit glucose uptake from the intestine and facilitate glucose disposal in the body [19]. In other words, the delay in pH adjustment in the gastrointestinal tract might have contributed to the delay in the digestive process responsible for the rise in blood glucose, thus suppressing the blood glucose increase. A study by Valls et al. reported that the optimal pH for α-amylase in saliva is pH 7–8, and that the activity of α-amylase decreases by approximately 30% at pH 5. The pH of gold kiwifruit was less than pH 4 in the PK and WK, and that of bread consumed in the BPK and BWK groups was also acidic (pH < 4). Therefore, the simultaneous consumption of gold kiwifruit with bread possibly attenuated the activity of α-amylase in saliva and suppressed oral digestion. We predicted that pre-consumption of gold kiwifruit would suppress the postprandial blood glucose elevation more effectively than simultaneous intake; however, the rate of ΔBGmax attenuation was 39.0% and 33.4% in the PK30B and WK30B groups, respectively, with no significant difference compared to that from simultaneous intake. The reason behind this outcome might have been the suppression of oral digestion in the BPK and BWK groups. It was suggested that the simultaneous intake of gold kiwifruit and bread (BKP and BWK groups) had the same inhibitory effect on blood glucose as prior intake of gold kiwifruit (KP30B and WK30B groups). In addition, GR measurements showed that the GR values in the BPK and BWK groups were almost similar to those of group B, and the results are displayed in Table 5. GR levels can predict the postprandial glycemic responsiveness of foods and meals [10], and the present results suggest that gold kiwifruit does not affect the rate of digestion in the small intestine.

In general, fruit pericarps contain polyphenols that inhibit α-glucosidase, and apples, for example, are known to inhibit the postprandial blood glucose elevation [20]. Adyanthaya et al. conducted an experiment using four types of apples (Cortland, Macintosh, Empire, and Mutsu) and discovered that the polyphenol content of the pericarp was higher than that of the flesh in all the varieties [12]. The polyphenol content was proportional to the alpha-glucosidase inhibitory effect, and McIntosh pericarp reportedly inhibited alpha-glucosidase by 75%. Since polyphenols are also present in gold kiwifruit pericarp, the effect was predicted to be consistent with that of apples. However, no significant difference in postprandial blood glucose levels was observed between the gold kiwifruit pericarp and pulp (BWK group) and gold kiwi pulp only (BPK group). Ingested carbohydrates are absorbed from the intestinal tract, transported through the hepatic portal vein to the liver, and subsequently to the rest of the body. After being supplied as an energy source to each body tissue, excess carbohydrates are stored as glycogen in the liver and muscles. Glycogen in the liver supplies carbohydrates to all tissues in the body while maintaining a constant blood glucose level, and plays a physiological role not only as an energy source but also in maintaining various metabolic processes and functions of the liver. Fructose is useful to the body as an energy source, converted to glucose, and used in the synthesis of triglycerides. It is incorporated into the metabolism (glycolysis) of glucose; however, it follows different pathways in the muscle and liver [21]. When fructose flows directly into the liver and is metabolized, it does not increase the blood glucose level (i.e., blood glucose concentration); nevertheless, it is converted to triglycerides in the liver, thus increasing very-low-density lipoprotein levels and potentially causing hyperlipidemia. However, a precise experiment in which mice were excess-glucose- and excess-fructose-labeled with an isotope (carbon-13) recently demonstrated that fructose is not immediately sent to the liver for metabolism after absorption in the intestinal tract, as previously considered, but is almost entirely metabolized in the small intestine in a small-intestine-specific manner. Most fructose metabolism has been reported to occur in small intestinal cells, where it is initially phosphorylated by ketohexokinase, which is present in the small intestine, and subsequently flows to downstream pathways. In the blood glucose profile of the B, BPK, and BWK treatment groups in this study, the BPK and BWK treatment groups predominantly demonstrated suppression of the blood glucose elevation. We speculated that this phenomenon was due to the difference in carbohydrates contained in bread and kiwifruit. In other words, the results of kiwifruit intake suggest that fructose metabolism in kiwifruit, unlike that of glucose, does not increase blood glucose directly, but is largely related to metabolism in small intestinal cells. Dietary fiber has been reported to suppress the postprandial blood glucose elevation and insulin sensitivity by delaying glucose absorption and promoting increased insulin sensitivity at the cellular level, thereby improving hyperglycemia [22]. In addition, kiwifruit contains soluble dietary fiber, which might have affected the suppression of the 30 min blood glucose increase in the PK30B and WK30B groups compared to that in the B group.

As shown in Figure 2, there was no significant difference in satiety between the groups 180 min after test-meal consumption. A satiety study by Lubransky et al. [12] demonstrated that there was no significant difference in satiety, even when 40% of the 65 g of carbohydrates in congee was replaced by kiwifruit, which was consumed 30 min before congee consumption. Our study hypothesized that there would be a difference in the blood glucose profile between subjects consuming kiwi with and without pericarps. However, the results of this study revealed that there was no difference between the effects of pericarp and those of no pericarp. The present study was conducted using the same amount of carbohydrates in each group. Interestingly, with the exception of group B, there was no difference in the level of satiety between the groups in the present study, which experienced a delayed gastric excretion rate due to the fructose and organic acids contained in kiwifruit. In other words, since there is no difference in satiety when the same level of carbohydrate content as that of bread is replaced with kiwifruit, we believe that consuming a meal in which a portion of the carbohydrate content (50 g) of starchy bread is replaced with kiwifruit will suppress the blood glucose elevation and contribute to people’s health as an idea for daily dietary guidance. This suggests that the quality of the carbohydrates contained in each food is a major factor in the suppression of elevated blood glucose levels. In the future, research that clarifies the effects of different carbohydrates contained in the bread and kiwifruit used in this study is imperative.

Since the present study was conducted on healthy adults, further investigation to determine whether the same effect can be obtained in patients with diabetes is warranted. Finally, gold kiwifruit can be cut in half and consumed comfortably. It is rich in nutrients, such as vitamin C and dietary fiber, and is considered an ideal fruit not only for diabetes prevention, but also for people in modern society who are too busy to obtain sufficient nutrition. The study reported results of a very small number of people, who were healthy and did not have any previous health issues. Therefore, in the case of actual diabetic disease, one has to take into account that there are also dietary and caloric restrictions. However, considering the preventive aspect, we believe that this study is a reported case that can contribute to healthy life expectancy.

## 5. Conclusions

This study demonstrated that gold kiwifruit, with or without pericarp, significantly suppressed the postprandial blood glucose elevation without decreasing satiety, regardless of the intake timing.

## Figures and Tables

**Table 1:** Subject characteristics.

	Total (*n* = 12)	Male (*n* = 6)	Female (*n* = 6)
Age (year)	24.8 ± 8.2	22.5 ± 0.8	27.0 ± 11.2
Height (cm)	165.6 ± 8.9	172.3 ± 4.6	158.9 ± 6.9
Weight (kg)	56.8 ± 6.9	60.7 ± 4.6	52.8 ± 6.4
BMI (kg/m^2^)	20.7 ± 1.9	20.5 ± 1.3	20.9 ± 2.0
Hb Alc (%)	5.2 ± 0.2	5.3 ± 0.2	5.2 ± 0.2

Values are shown as mean ± S.D.

**Table 2:** Nutritional components and test-food amounts.

	B	PK	WK
Weight (g)	100.0	100.0	100.0
Height (cm)	250.7	62.2	68.0
Protein (g)	8.5	0.9	0.9
Fat (g)	2.8	0.2	0.2
CHO A vail. (g)	45.9	13.4	14.5
Fiber (g)	(2.3)	1.4	2.2

B: Bread, PK: Pulp of Kiwi, WK: Whole Kiwi. B: Bread * URL: https://www.fujipan.co.jp/component/02.html (japanese) (accessed on 19 June 2021). K: kiwi * URL: https://www.zespri.com/ja-JP/blogdetail/8-important-nutrients (accessed on 19 June 2021).

**Table 3:** Kinetic parameters of ΔBlood Glucose levels. Values are presented as mean ± SEM (*n* = 12).

	ΔBG_max_ (mg/dL)	ΔT_max_ (min)	IAUC (mg·min/dL)
B	67.4 ± 4.2	48.8 ± 4.4	4267.0 ± 397.5
BPK	49.3 ± 5.5 *	39.6 ± 2.9	2955.9 ± 326.3 *
BWK	49.6 ± 5.2 *	38.8 ± 3.3	2074.1 ± 288.6 **
B30B	43.5 ± 4.6 **	70.0 ± 7.4	3194.8 ± 426.6
PK30B	40.2 ± 2.9 **	70.0 ± 6.9	2259.4 ± 288.8 **
WK30B	44.3 ± 4.0 **	76.3 ± 6.0	3256.9 ± 403.5

Values are shown as mean ± SEM (*n* = 12). *: *p* < 0.05 vs. B, **: *p* < 0.01 vs. B *(Dunnett’s test).*

**Table 4:** Suppression rate of ΔBlood Glucose levels.

	ΔBG_max_	IAUC
BPK	−27.6% ± 4.8	−29.3% ± 5.8
BWK	−25.1% ± 7.0	−46.2% ± 7.4
B30B	−35.2% ± 5.6	−19.4% ± 10.8
PK30B	−39.0% ± 4.3	−45.0% ± 7.3
WK30B	−33.4% ± 6.1	−15.6% ± 14.4

Values are shown mean ± SEM, *n* =12.

**Table 5:** Glucose releasing rate of test foods. Values are presented as mean ± SEM (*n* = 6).

Materials	Glucose Concentration (µg/mL)		GR
	20 min	16 h	
PK	151.2 ± 2.3	153.9 ± 2.4	984 ± 2.6
WK	147.5 ± 1.4	145.5 ± 1.3	101.3 ± 1.7
B	265.4 ± 5.9	338.7 ± 4.8	78.3 ± 1.0
BPK	332.9 ± 8.9	430.0 ± 5.6	77.5 ± 2.0
BWK	282.8 ± 4.1	337.7 ± 1.8	83.8 ± 1.5

Values are shown as mean ± SEM (*n* = 6). There was no significant difference in each sample *(Dunnett’s test).*

## Data Availability

Data sharing not applicable.

## Supplementary Materials

The following are available online at https://www.mdpi.com/article/10.3390/nu13062103/s1, Table S1: subject characteristics (N = 12).

## Abbreviations

CHO	carbohydrate
GI	glycemic index
GR	Glucose Releasing Rate
Group B	bread
BPK group	simultaneous consumption of B (79.9 g) and gold kiwifruit pulp (PK, 100 g)
BWK group	simultaneous consumption of B (79.9 g) and gold kiwi pericarp/pulp (WK, 92.4 g)
B30B group	consumption of B (79.9 g) 30 min after ingesting B (29.2 g)
PK30B group	consumption of B (79.9 g) 30 min after ingesting PK (100 g)
WK30B group	consumption of B (79.9 g) 30 min after ingesting WK (92.4 g)
