# Supplementary material for: The Effects of Gold Kiwifruit Intake Timing with or without Pericarp on Postprandial Blood Glucose Level
Source: Nutrients. 2021 Jun 19;13(6):2103. doi: 10.3390/nu13062103 (PMC8235107; doi:10.3390/nu13062103)
Supplement: Supplementary file 1 [file nutrients-13-02103-s001.zip › nutrients-1194898-SI.pdf]

Table SI 1 Subject characteristics (N=12)

Subjects	A	B	C	D	E	F	G	H	I	J	K	L
Male /Female	M	M	M	M	M	M	F	F	F	F	F	F
Age (year)	23	23	23	23	21	22	21	23	22	22	22	52
Height (cm)	164.3	169.0	174.0	178.1	171.8	176.4	164.1	163.0	148.1	158.4	167.5	152.0
Weight (kg)	55.4	57.5	70.0	62.0	59.5	60.0	54.6	55.0	38.6	55.2	57.5	56.0
BMI (kg/m^2)	20.52	20.13	23.12	19.55	20.16	19.28	20.28	20.7	17.6	22.0	20.49	24.24
HbA1c (%)	5.2	5.4	5.6	5.5	5.0	5.2	5.2	5.4	5.1	4.9	5.0	5.4
